# Association Between Joint Physical Activity and Dietary Quality and Lower Risk of Depression Symptoms in US Adults: Cross-sectional NHANES Study

**DOI:** 10.2196/45776

**Published:** 2023-05-10

**Authors:** Jinghong Liang, Shan Huang, Nan Jiang, Aerziguli Kakaer, Yican Chen, Meiling Liu, Yingqi Pu, Shaoyi Huang, Xueya Pu, Yu Zhao, Yajun Chen

**Affiliations:** 1 Department of Maternal and Child Health, School of Public Health, Sun Yat-sen University Guangzhou China

**Keywords:** physical activity, dietary quality, depression symptom, adults, NHANES

## Abstract

**Background:**

Depression escalating public health concern and the modest efficacy of currently available treatments have prompted efforts to identify modifiable risk factors associated with depression symptoms. Physical inactivity, poor nutrition, or other lifestyle behaviors are among the potentially modifiable risk factors most consistently linked with depression. Past evidence regarding the single effect of physical activity (PA) or dietary quality (DQ) on reducing the risk of depression symptoms has been well-documented. However, the association of the joint effect of PA and DQ on depression symptoms has never been investigated in a representative sample of adults.

**Objective:**

This study investigates the association between PA and depression symptoms and between DQ and depression symptoms, and their combined effects on US adults.

**Methods:**

Data were obtained from the National Health and Nutrition Examination Survey (NHANES) 2007 to 2018 cycles. The primary exposures were DQ and PA, measured using the Healthy Eating Index (HEI)-2015 and the metabolic equivalent (MET) minutes per week reported in questionnaires, respectively. Depression symptoms were defined as a 9-item Patient Health Questionnaire (PHQ-9) score of ≥10. We created 4 lifestyle categories: healthy diet and active individuals, unhealthy diet but active individuals, healthy diet but inactive individuals, and unhealthy diet and inactive individuals. Participants were considered to have a healthy diet if they fell within the 60th percentile of the HEI-2015 or to be active if they met the current guidelines for PA. A survey-multivariable logistic regression approach was used to model adjust the variables relevant to the associations, and an age-adjusted prevalence for depression symptoms was calculated following the NHANES guidelines.

**Results:**

In total, 19,295 participants represented a weighted number of 932.5 million adults aged 20 to 80 years in the noninstitutionalized US population. The total age-adjusted prevalence of depression symptoms among all respondents was 7.08% (1507/19,295). Of the respondents, 81.97% (15,816/19,295) met the PA recommendation and 26.79% (5170/19,295) scored at or above the 60th percentile on the HEI-2015. Depression symptoms were inversely associated with a higher level of PA (adjusted odds ratio [AOR] 0.819, 95% CI 0.716-0.938) and healthy DQ (AOR 0.809, 95% CI 0.701-0.931), respectively. A healthy diet combined with recommended PA was associated with a significantly lower risk of depression symptoms (AOR 0.658, 95% CI 0.538-0.803) than those who consumed an unhealthy diet but were physically active (AOR 0.890, 95% CI 0.765-1.038) or consumed a healthy diet but were physically inactive (AOR 1.077, 95% CI 0.817-1.406).

**Conclusions:**

Our findings indicate that people with a healthy diet and recommended PA have a lower risk of depression symptoms than those with an unhealthy diet and a low level of PA. A healthy dietary habit and regular PA are potential precautions against depression.

## Introduction

Depression, a major worldwide public health concern commonly characterized by low emotion, diminished interest, and decreased energy and attention, has been affecting nearly 7.2% to 9.2% of the US population, particularly adults, from 2015 to 2020 [1]. Depression will continue to affect individuals, the health care system, and the whole society if left unprevented [2]. The growing prevalence of depression poses an increasing demand for highly effective, preventative approaches. Some studies have reported potentially protective effects of dietary quality (DQ) or physical activity (PA) on depression symptoms, as well as the biological actions of nutrients or behavior, both within and beyond the context of healthy behavior [3,4]. Understanding PA and DQ is vital to intervene against depression symptom risks during adulthood, as dietary pattern, PA, and other behaviors are the major triggers determining the well-being of mental health [5].

Nevertheless, robust evidence concerning the association between the single effect of DQ or PA and the prevalence of depression symptoms remains sparse and debated. For example, although PA was linked to a decreased risk of depression in several studies [6,7], these findings were not consistently replicated in another study [8]. Moreover, little is known about the combined influence of dietary behaviors and PA on depression symptoms in the US population. It can be argued that healthy diet and sufficient activity may be more effective than unhealthy diet and inactivity in promoting the mental health of adults. Nevertheless, empirical evidence for it is far from clear cut, and there exist limitations in prior related studies [9-14]. First, most previous studies simply examined isolated nutrients, such as energy intake and eating behavior, for depression symptoms, rather than the total DQ [9,10], which is a comprehensive representation of an individual’s overall diet pattern. Second, the most important finding is that no studies so far have specifically targeted the association between DQ and PA and depression symptoms among the US population, except for some studies that investigated this exposure on other health indicators [11-13]. Furthermore, a few studies reported the trends for DQ with serial cross-sectional investigations using older data (updated to the year 2016) [14], but these findings need to be verified by more subsequent national data (National Health and Nutrition Examination Survey [NHANES] is updated to the 2020 cycle). Finally, as there might be no race-related information available in their studies, the impact of discrepancy on race or ethnicity on diets, which deserves more attention, was often neglected in previous studies. For example, Black people, commonly reported to have poor DQ, have the highest morbidity rates of diet-related diseases in comparison with other racial or ethnic groups in the United States [15,16]. Individual depression symptoms might be affected by lifestyle, depending on sex, age, and race, but no relevant study has examined the heterogeneity of populations. To compensate for these limitations, we sought to describe the prevalence of depression symptoms and its association with DQ and PA, considering age, race or ethnicity, sex, and sociodemographic status, among the US population using data from the continuous NHANES.

## Methods

### Study Design

**Figure 1:**
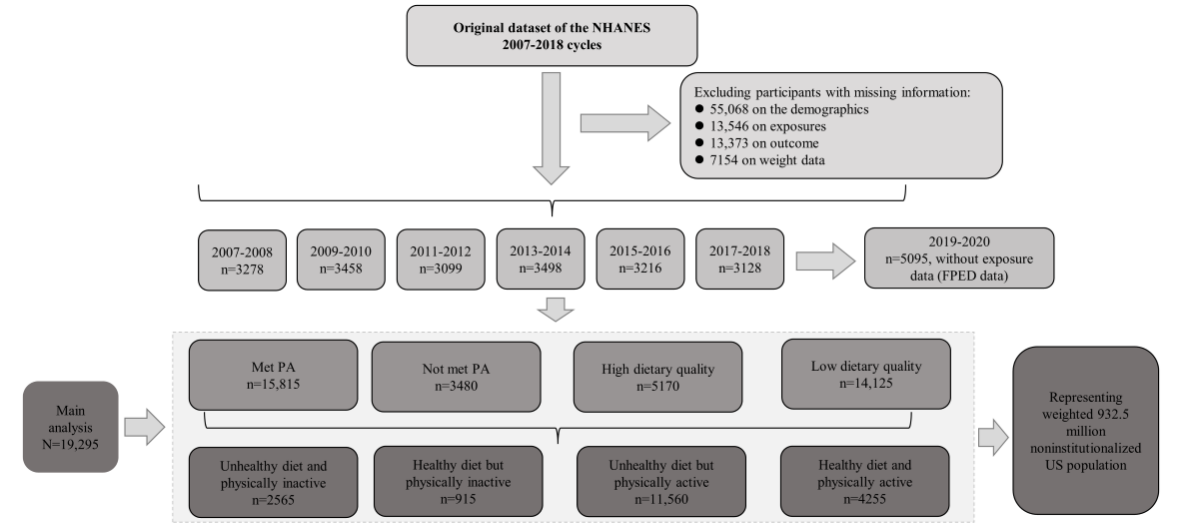
Flowchart of selection of National Health and Nutrition Examination Survey (NHANES) participants: unhealthy diet and physically inactive individuals (participants did not meet the US physical activity [PA] recommendation guideline and below the 60th percentile of the Healthy Eating Index [HEI]-2015 score), healthy diet but physically inactive individuals (participants did not meet the US PA recommendation guideline but at or above the 60th percentile of the HEI-2015 score), unhealthy diet but physically active individuals (participants met the US PA recommendation guideline but below the 60th percentile of the HEI-2015 score), healthy diet and physically active individuals (participants met the US PA recommendation guideline and at or above the 60th percentile of the HEI-2015 score). FPED: food patterns equivalents database.

The NHANES is an ongoing cross-sectional program conducted by the National Center for Health Statistics of the Centers for Disease Control and Prevention to investigate the public’s general health-related behaviors, their socioeconomic and nutritional status, and the results of physical examinations. With a multistage, stratified probability sample selected from cities, blocks, households, and the number of people within households, the NHANES offers comprehensive data to represent the US civilian and noninstitutionalized population. The objectives, study design, and implementation of the NHANES have been introduced before [14]. The 24-hour dietary recall was administered in person by a trained interviewer using the United States Department of Agriculture automated multiple-pass method [17]. This study was conducted in accordance with the Declaration of Helsinki [18]. Flowchart of selection of NHANES participants can be found in Figure 1.

### Ethics Approval and Informed Consent

Written informed consent was obtained from all study participants, and the program was approved by the Ethics Review Board of the National Center for Health Statistics [19]. The NHANES data are free for public use and available on the web [20], so it was not necessary to obtain the agreement of the medical ethics committee board [21].

### Assessment for DQ

With an interval of 3 to 10 days, two 24-hour recalls of all the food and drink consumed the day before the interview (midnight to midnight) were conducted. The Healthy Eating Index (HEI)-2015 is a comprehensive measurement of an individual’s dietary pattern with overall and subcomponents scores, which adheres to the 2010 Dietary Guidelines for Americans [22]. The HEI-2015 contains 13 nutrient- and food-based components, which comprise 9 adequacy food components (total fruits, whole fruits, total vegetables, greens and beans, whole grains, dairy, total protein foods, seafood and plant proteins, and fatty acids) and 4 moderation components (refined grains, sodium, added sugars, and saturated fats). The total HEI-2015 score ranges from 0 to 100, with a higher score indicating greater overall DQ [22]. Participants whose average score of the 2 days was in or above the 60th percentile in the HEI were considered to have observed the dietary guidelines or consumed healthy foods [23].

### Assessment for PA

Data were collected using the Global Physical Activity Questionnaire. Created by the World Health Organization (WHO), the Global Physical Activity Questionnaire was used to assess different domains of individuals’ PA, such as leisure-time PA, occupation, and transportation PA [24,25]. In accordance with the WHO analysis guide, PA was converted to metabolic equivalent (MET) minutes of moderate to vigorous PA per week [24]. MET values vary with the type of exercise, and the NHANES offers the recommended MET values for each PA. PA was based on the MET values of type, frequency, and duration of activities per week, which was calculated using the following formula: PA (MET-min/wk) = MET × weekly frequency × duration of each PA [26]. PA=0 denotes participants who do not engage in any PA, else, it means that participants have constant or intermittent PA. The respondents were classified based on whether they met the American PA guideline (PA at moderate intensity should be done for 150 minutes [equivalent to 600 MET min/wk] a week or, at vigorous intensity, should be performed 75 min/wk for adults [27]).

### Definitions of Lifestyle Groups for PA and DQ

According to previous studies [13,23], we created four lifestyle categories according to DQ and PA: (1) healthy diet and active individuals, (2) unhealthy diet but active individuals, (3) healthy diet but inactive individuals, and (4) unhealthy diet and inactive individuals. Participants were considered to have a healthy diet if they fell within the 60th percentile of the HEI or were active if they met the current guidelines for PA [13,27].

### Outcome Measurements

The 9-item Patient Health Questionnaire (PHQ-9) is a brief self-report measure of depression symptoms in a primary care and research setting that has a well-established factor structure, reliability, and validity [28,29]. The range of scores for PHQ-9 is from 0 to 27, with higher scores reflecting greater severity, and a PHQ-9 score of ≥10 was recommended as the binary threshold to define the presence of depression symptoms, which has a sensitivity of 88% and a specificity of 88% for screening major depression symptoms [30].

### Assessment of Covariates

According to prior research and clinical experts, potentially confounding and modifying variables were identified [31] as follow: age group (20-39, 40-59, or 60-80 years), sex (male or female), race or ethnicity (non-Hispanic White, non-Hispanic Black, Mexican American, Other races [including multiracial and other Hispanic]), the highest level of education achieved (less than 9th grade, 9th-11th grade [including 12th grade without diploma], high school graduate [general educational development or equivalent], college graduate or above, or some college or associate’s degree), marital status (widowed or divorced or separated, never married, married or living with partner), poverty-to-income ratio (PIR; a family poverty index was used to estimate socioeconomic status, which is calculated by measuring the income thresholds of different types of households and updating them annually because of inflation, based on the Consumer Price Index. According to the commonly used cutoff levels for identifying eligibility for federal assistance, we classified these ratios as being either ≤100% or ≥100% of the poverty threshold, depending on whether they exceeded the poverty threshold), BMI (calculated based on the ratio of weight in kilograms to height in meters squared), smoking status (current, previous, or never), whether taking antidepressant or anxiolytic medications, sleep time (<7 h, 9 h, and >9 h), and sedentary behavior (SB; the amount of time spent sitting per day is considered SB, including sitting at a desk reading; playing cards; watching television or using a computer; sitting with friends; and traveling by car, bus, or train).

### Statistical Analyses

We used weighted samples and considered stratification and clustering in the design to generate nationally representative estimates that were applied to US residents [32]. The combined NHANES cycle and weights were constructed following the guidelines for continuous NHANES analyses [33]. Proportional outcome variables are presented as means (SD) with analysis of variance F and the proportional outcome variables as proportions (%) with analysis of the chi-square test to determine statistical significance by 4 groups. The estimates obtained from adjusted odds ratios (AORs) and corresponding 95% CIs were used to evaluate the associations between PA and DQ and depression symptoms. A survey-multivariable logistic regression approach was used to adjust the variables relevant to the associations. For each lifestyle group, the median values of health variables were treated as continuous variables in a multivariate linear regression model to estimate the trend between each lifestyle and depression symptom. We used 3 different models: a crude model; a model adjusted for age, sex, and race or ethnicity; and a model in which we additionally adjusted for age, sex, race or ethnicity, education, marital status, BMI, PIR, smoking status, sleep time, and SB. We also generated 3 indicators along with their 95% CI, namely, relative excess risk due to interaction (RERI), attributable proportion due to interaction (AP), and synergy index (SI), to estimate the interactive effect between PA and DQ [34,35]. The 95% CI of RERI and AP containing 0 indicates there is an additive interaction between the 2 exposures, and the greater the absolute value of RERI, the stronger the interaction between the 2 exposures (PA and DQ) [36]. For a 95% CI of SI, if it contains 1, it indicates that there exists an additive interaction between the 2 exposures (PA and DQ) simultaneously. An SI value greater than 1 indicates that the synergistic effect is enhanced when the factors were both exposures, while a value below 1 indicates that the effect of the 2 factors is weakened [36]. An age-adjusted prevalence for depression symptoms was calculated according to the NHANES guidelines [37]. To account for potential variability in the associations in terms of age, sex, and other demographic characteristics, exploratory subgroup analyses were performed with the interactions obtained, followed by sensitivity analyses after excluding individuals exposed to extreme PIR or individuals with extreme BMI values to identify the robustness of our findings. Moreover, alcohol use (former, never, and now), total energy, and whether taking antidepressant or anxiolytic medications were regarded as additional covariates to control their potential influence. The probability of the null hypothesis value (*P* value) below .05 was considered to reflect a statistically significant difference. All statistical analyses were conducted using R statistical programming language (X64 version 4.1.0; R Foundation for Statistical Computing).

## Results

### General Characteristics

**Figure 2:**
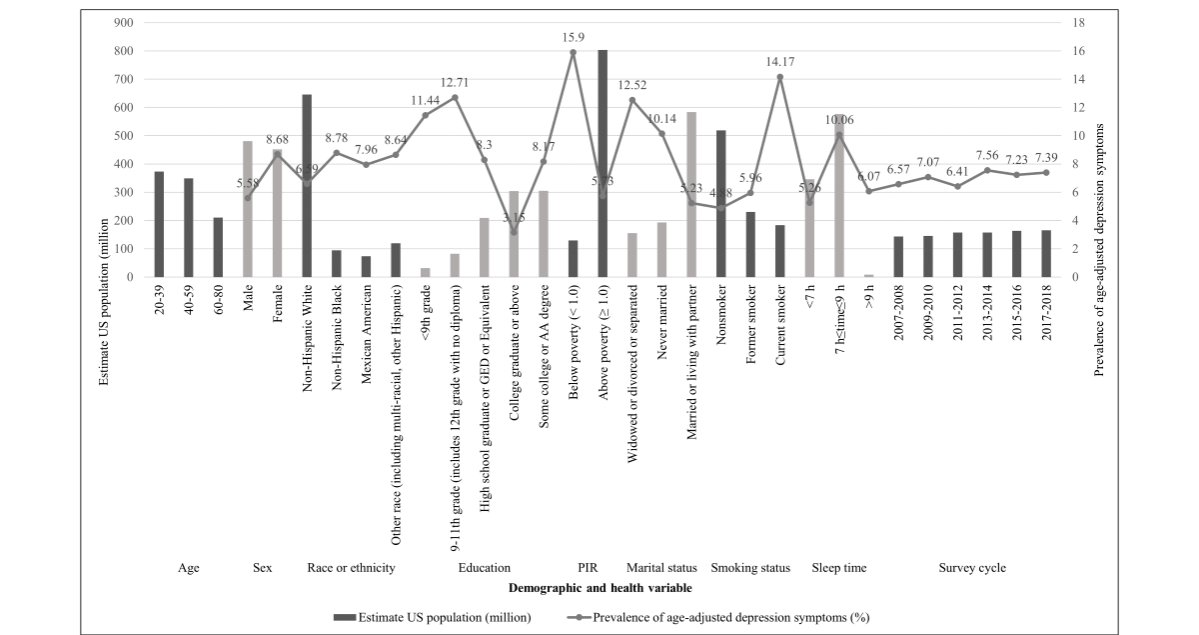
Prevalence of age-adjusted depression symptoms and estimate US population of different characteristics among US general adults, National Health and Nutrition Examination Survey 2007 to 2018. AA: Associate’s Degree; GED: general educational development; PIR: poverty income ratio.

Approximately, a weighted population of 932.5 million American residents was represented by 19,295 NHANES participants with valid data, and most of them were non-Hispanic White participants (645.6 million; Multimedia Appendix 1). Among the 19,295 respondents, 52.59% (n=10,147) were female, 44.76% (n=8637) were non-Hispanic White, 80.54% (n=15,541) had at least a high school education, and 20% (n=3859) lived in households with incomes below the federal poverty level. Overall, 81.97% (15,815/19,295) of the respondents met the PA recommendation, and 26.79% (5170/19,295) of participants scored at or above the 60th percentile on the HEI-2015. Moreover, participants with a healthy diet and regular exercise had an average HEI-2015 score of 69.32 (SD 0.19) and were active with 4476 (SD 105.15) MET minutes moderate to vigorous PA per week. In contrast, people who eat unhealthily and are inactive have an average score of 44.87 (SD 0.25) and only 316.90 (SD 4.24) minutes of moderate to vigorous PA per week. The age-adjusted prevalence of depression symptoms among respondents who did not meet the PA guidelines and had a low-quality diet was 9.51% (264/2565). In total, 4.19% (200/4255) of participants with depression symptoms in the group met the PA recommendations and had a higher DQ. The age-adjusted prevalence for another 2 groups was 7.54% (961/11,560; unhealthy diet but physically active) and 8% (82/915; healthy diet but physically inactive). We also found a relatively higher age-adjusted depression symptom prevalence in female (922/9148, 8.68%), low education participants (<9th grade: 152/1381, 11.44%; 9th-11th grade: 313/2373, 12.71%), and especially smokers (586/3982, 14.17%). Multimedia Appendix 1 and Figure 2 present the characteristics of the 19,295 US adults according to their lifestyles. The demographic characteristics and health variables of participants, both classified by depression symptom status and 6 survey cycles, are presented separately in Multimedia Appendices 2 and 3.

### Association Between Single PA or DQ and Depression Symptoms

**Figure 3:**
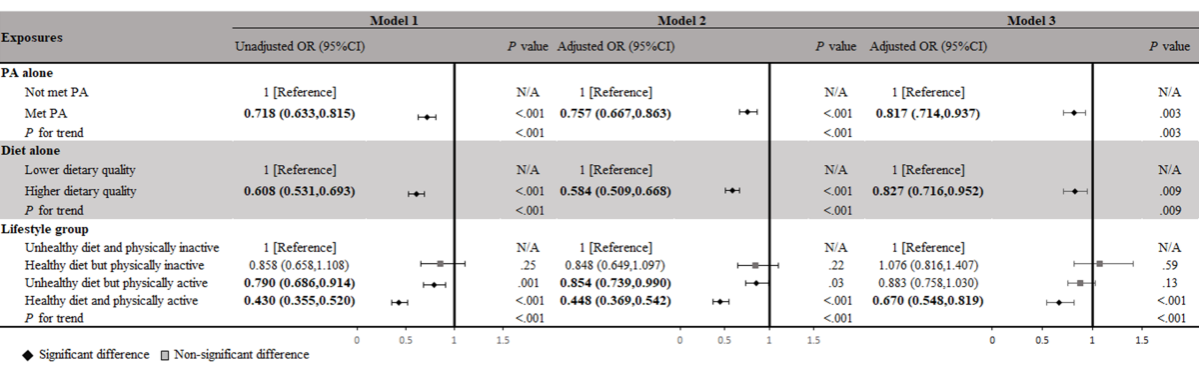
Association between PA and dietary quality and their combined effect and prevalence of depression symptoms among 19,295 study participants. Significant results are in bold. Model 1: crude model, no covariates were adjusted; model 2: adjusted for age, sex, and race or ethnicity; model 3: adjusted for age, sex, race or ethnicity, education, marital status, BMI, poverty-to-income ratio (PIR), smoking status, sedentary behavior (SB), and sleep time. N/A: not applicable; OR: odds ratio; PA: physical activity.

For the single effect of PA or DQ on depression symptoms, a statistically significant inverse relationship was found between the single PA effect and depression symptom prevalence in each model (*P*<.001; Figure 3). An inverse significant association was detected after controlling for multiple variables in the met PA guidelines group (AOR 0.817, 95% CI 0.714, 0.937; *P*=.003). Similarly, we noted a significant positive association between the single DQ effect and depression symptom prevalence: participants with a higher DQ had a lower risk of depression symptoms (AOR 0.827, 95% CI 0.716, 0.952; *P*=.009) compared with those with poor DQ (Figure 3).

### Joint Association Between PA and DQ and Depression Symptoms

Regarding the joint effect of PA and DQ on depression symptoms, when comparing depression symptom prevalence between groups with different lifestyles, participants consuming a healthier diet and being more physically active had a lower risk ratio of depression symptoms of 0.670 (95% CI 0.548-0.819; *P*<.001). Participants who consumed a healthy diet but were physically inactive did not have a significantly lower risk of depression symptoms (AOR 1.076, 95% CI 0.816-1.407; *P*=.59), as well as those who consumed an unhealthy diet and were physically active (AOR 0.883, 95% CI 0.758-1.030; *P*=.13). The results regarding the additive interaction between the 2 exposures (PA and DQ) showed that the RERI and AP between PA and DQ were statistically significant (RERI=−0.052, 95% CI −2.149 to 2.044; AP=−0.078, 95% CI −3.333 to 3.177), which meant there is an additive interaction between the 2 exposures. A similar significant interactive effect of SI (SI=1.189, 95% CI 0.001-1170.880) was observed, which indicates the synergistic effect of PA and DQ is enhanced in our model (Figure 3).

### Subgroup Analyses and Sensitivity Analyses

**Figure 4:**
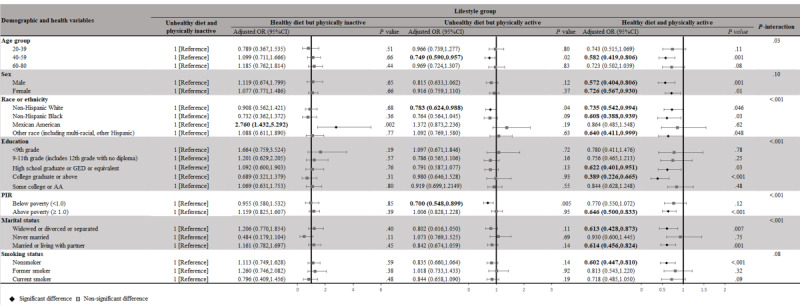
Associations between exposure to different lifestyle groups and incidence of depression symptoms in stratified analyses. Significant results are in bold. The multivariable model was adjusted for age, sex, race or ethnicity, education, marital status, BMI, PIR, smoking status, SB, and sleep time. AA: Associate’s Degree; GED: general educational development; OR: odds ratio; PIR: poverty-to-income ratio; SB: sedentary behavior.

The main analysis of the lifestyle-depression symptom association based on demographic subgroups showed similar AORs within each group (Figure 4). The AORs remained significant for participants having a healthy diet and PA, who were male (AOR 0.572, 95% CI 0.404-0.806; *P*<.001), who were non-Hispanic Black (AOR 0.608, 95% CI 0.388-0.939; *P*<.05), or had a PIR above the average level (AOR 0.646, 95% CI 0.500-0.833; *P*<.001). In the sensitivity analyses for depression symptoms attributed to lifestyle exposures, after further adjusting for several potential indicators (alcohol users [former, never, and now], total energy, whether taking antidepressant or anxiolytic medications, and sleep time), the results remained similar in that DQ and PA were positively correlated with mental health (Multimedia Appendix 4).

## Discussion

### Principal Findings

Comprising 19,295 participants with 6 waves (NHANES cycles: 2007-2018), the overall analysis indicated that lower depression symptom rates were seen in participants who either followed a healthier dietary pattern or were engaged in more PA and who both had better DQ and more PA. The association remained significant when other potential covariates were controlled for in sensitivity analyses.

The benefits of a healthy diet or PA alone for depression have been well-documented [3,38]. However, data from the latest Global Status Report on PA issued by the WHO in October 2022 showed that 27.5% of adults failed to meet the WHO’s recommendation for PA that an adult should work out 150 to 300 minutes a week with moderate PA or 75 to 150 minutes a week at an aerobic intensity [27]. Consequently, among the 27.5% of adults with insufficient PA, 47% had health-related indicators of noncommunicable diseases caused by hypertension, and 43% had depression [39]. Similarly, our results also showed that 18.03% of US adult participants did not have adequate PA, as recommended by the WHO. The similarity of sample characteristics between our study and the global report makes our findings more reliable and increases their authenticity. In line with previous studies, our results showed that a single effect of PA or DQ was significantly associated with mental health [40,41]. This association is explained by the reaction of the endocannabinoid system to acute neuroendocrine stimulation and inflammation [42] and long-term brain adaptations, such as changes in neural architecture [43]. A Mendelian randomization study has investigated more than 100 potentially modifiable factors for their association with incident depression, but it only identified several modifiable risk factors for the prevention of depression, including PA-related domains and dietary [44]. PA showed a potential causal connection with depression in a large-scale genome-wide association study, which suggested more PA might help prevent depression [45].

### Comparison With Prior Work

As noted earlier, there might exist a causal relationship between dietary habits and depression [44]. A comprehensive food-based diet pattern made up of multiple dietary components or food groupings, rather than single nutrients or foods, has become widespread and helps us understand health trajectories, as such dietary patterns can evaluate the interplay between nutrients and foods consumed simultaneously through a multidimensional approach with the impact of the overall diet considered [46,47]. Our findings revealed that a healthy diet was negatively associated with depression symptoms among adults by comparing their DQ. Recent studies using the same NHANES data to investigate the association between HEI and depression symptoms concluded that higher HEI scores significantly correlated with fewer depression symptoms [48]. For all age groups, a constantly optimized diet will probably offer substantial health gains, which are predicted to be larger with earlier optimization in diet [49]. The hypotheses for healthy dietary pattern are also applicable to the prevention of depression symptoms, probably because an inflammatory process can be inhibited by a high-quality diet that includes vegetables, fruits, whole grains, nuts, and legumes [50,51]. Several inflammatory markers, namely IL-1, IL-6, TNF-α, and hsCRP, have been found to be elevated in individuals with depression [52], and thus depression may be pathophysiologically underpinned by chronic inflammation. The researchers also found that people who closely followed the specific dietary patterns, such as the Dietary Approaches to Stop Hypertension (DASH) diet, Mediterranean diet, or Mediterranean-DASH Intervention for Neurodegenerative Delay diet—a newly developed one that incorporates elements from the Mediterranean and DASH diets, emphasizes plant-based food, and avoids animal-based food—tended to have lower depression risk [4,45,46]. Among the recommended diet for reducing depression risk are fruits, vegetables, legumes, wholegrain cereals, and nuts instead of unhealthy products such as fast foods, pastries, and soft drinks [53]. Despite the fact that existing evidence on DQ only discussed certain kinds of food, the healthy dietary pattern in our study is comprehensive enough to cover the aforementioned nutrients, whose potential positive impact on depression symptoms is worthy of prompt replication in larger populations [54]. As well-documented as either the single effect of diet or PA alone for preventing depression is, there are no relevant studies that have examined the effects of diet and PA as a whole on depression. PA and a reduction in certain “unhealthy” foods, such as fast foods and pastries, are widely recognized as independently protective against depression. However, these studies used self-reported PA measures and focused on a single nutrient or nutrient group rather than on the quality of the overall diet. Our study provides a new comprehensive diet pattern for depression prevention unlike previous findings focusing on single nutrients or food groups. A comprehensive DQ score captures not only individual intake levels but also higher-level interactions. Meanwhile, our results showed that PA exposure was also a recommended lifestyle. Specifically, this study emphasizes the importance of incorporating dietary behaviors into health behavior models when examining influence of PA on depression. On the basis of the most recent NHANES data (updated to the 2018 cycle) with 19,295 participants on behalf of approximately 932,548,888 US adults, the study measured health parameters for each of the 4 lifestyle groups to further demonstrate the joint benefits of adequate PA and healthy diet for depression symptoms among US adults. Furthermore, to enhance the robustness of our analyses, we have also considered a moderate number of potential confounders, such as SB, an important factor related to individual health that is independent of PA. On the basis of the findings of the integrated effect of PA and DQ on depression symptoms, we found that the risk of depression symptoms had reduced for participants who achieved the recommended PA level or consumed a healthy diet. However, when participants with different PA levels and DQ were classified into 4 lifestyle groups to examine the combined effect of PA and DQ, correlations of the 2 lifestyles (unhealthy diet but physically active and healthy diet but physically inactive) with depression symptoms (reported in Figure 3) were mild and moderate, respectively, without statistical significance. It showed that the combination of PA and DQ may be more effective than either factor alone in reducing depression symptoms. Hence, it may be hypothesized that regular PA and a healthy diet are both at work for the prevention of depression symptoms and neither can be omitted. In addition, our model has further demonstrated that there existed an additive interaction between PA and DQ, and the synergistic effect is enhanced on the improvement of depression symptoms when the recommended PA level and a healthy diet are simultaneously exposed. Such a synergistic effect may be linked to the Lifestyle Medicine (LM) preventive strategy, a multicomponent and multimodal therapeutic approach that combines nutrition, PA, and other behaviors management [55]. That the pathogenesis and progression of depression involve lifestyle determinants has been well established, although the effectiveness of these determinants in helping patients with depression is underappreciated in current first-line treatments [56]. The analysis of the integrated effect in our study agreed with the results of the meta-analysis that examined the effect of multicomponent LM interventions, which include PA, DQ, and smoking cessation, on relieving depression symptoms (nonclinical depression) among adults in comparison with the named control group [5]. This study deliberately excluded patients with clinical depression to examine the preventive effect of LM intervention on depression symptoms [5]. The results highlighted that patients with depression might benefit greatly from the LM intervention, whose potential effect in the prevention of depression symptoms may be valued by major clinical guidelines and mental health experts [57]. One meta-analysis analyzed 50 randomized controlled trials concerning LM intervention and revealed that the effect of multicomponent LM intervention on depression symptoms was greatly moderated by the number of lifestyle factors adopted [58], which was consistent with our main finding that the synergistic effect was enhanced when more lifestyle behaviors that are beneficial to depression symptoms have been involved in one’s daily life. Considering that the multicomponent LM intervention could possibly be used in clinical practice of controlling depression, examining the preventative effect of this intervention on depression symptoms is therefore essential. LM may be a promising complementary treatment for depression symptoms. However, more research is needed to determine its effectiveness and applicability as an adjunct to existing first-line treatments and to understand the impact of LM interventions on different populations, such as adolescents and older adults [59]. On the basis of these findings, it is important to combine DQ and PA in national and community strategies for depression symptoms to obtain the optimal prevention effects.

### Limitations

However, we acknowledge the possibility of limitations in our study that must be considered. First, PA was only measured by self-report, which is subject to recall and social desirability biases. Second, our cross-sectional study design made it difficult to establish causal links between diet and PA and depression. Nevertheless, evidence regarding the single effect of PA or a healthy diet on depression symptoms has been identified in previous Mendelian randomization studies [44,45], implying that reverse causation was less likely to occur in our study. Thus, high-quality prospective population-based studies should be conducted to verify this causal relationship. Third, as another common depression symptom population, children and adolescents are not included as they have not completed the relevant assessment concerning PA and depression symptoms. Thus, caution should be exercised when extrapolating this association in general adults to younger populations. Fourth, several potential risk factors, such as hypertension and diabetes mellitus, may also be the cause of the depression symptoms but are not considered as covariates in our study. Moreover, as our study focused on the PA pattern, we did not further explore the association between PA frequency, duration, and intensity and depression symptoms, and the dose-response relationship between them, which could be examined in a future study.

### Conclusions

On the basis of the NHANES data from the 1999 to 2018 cycle, our study found that a higher level of PA incorporated with better DQ was inversely related to depression symptoms in a representative sample of the US population, which meant that participants experiencing the recommended PA level and consuming a healthy diet together enhance the synergistic effect on depression symptoms. Therefore, our findings help identify the elements of specific lifestyles that are effective for the prevention of depression symptoms and offer useful reference for decision makers to start relevant preventive programs and promote them to the public.

## Appendix

Multimedia Appendix 1 Adjusted weighted characteristics of 19,295 US adults ≥20 years, stratified by lifestyle groups, the National Health and Nutrition Examination Survey 2007 to 2018.

Multimedia Appendix 2 Characteristics of 19,295 US adults ≥20 years, stratified by depression symptom status, the National Health and Nutrition Examination Survey 2007 to 2018.

Multimedia Appendix 3 Characteristics of 19,295 US adults ≥20 years, stratified by survey cycle, the National Health and Nutrition Examination Survey 2007 to 2018.

Multimedia Appendix 4 Association between lifestyle and risk of depression symptoms with the exclusion of extreme poverty-to-income ratio (PIR), BMI values, and adjustment for alcohol use, total energy intake, and use of antidepressant or anxiolytic medications.

## Abbreviations

AOR: adjusted odds ratio
AP: Attributable Proportion due to interaction
DASH: Dietary Approaches to Stop Hypertension
DQ: dietary quality
HEI: Healthy Eating Index
LM: Lifestyle Medicine
MET: metabolic equivalent
NHANES: National Health and Nutrition Examination Survey
PA: physical activity
PHQ-9: 9-item Patient Health Questionnaire
PIR: poverty-to-income ratio
RERI: Relative Excess Risk due to Interaction
SB: sedentary behavior
SI: Synergy Index
WHO: World Health Organization

## Data Availability

The data sets generated and analyzed during this study are available from the National Health and Nutrition Examination Survey (NHANES) databases 2007-2018 [60]. The data sets generated or analyzed during this study are available from the corresponding author upon reasonable request.
