# Supplementary material for: Association Between Joint Physical Activity and Dietary Quality and Lower Risk of Depression Symptoms in US Adults: Cross-sectional NHANES Study
Source: JMIR Public Health Surveill. 2023 May 10;9:e45776. doi: 10.2196/45776 (PMC10209797; doi:10.2196/45776)
Supplement: Multimedia Appendix 1 [file publichealth_v9i1e45776_app1.docx]

**Table S1** Adjusted weighted characteristics of 19,295 US adults ≥20 years, stratified by lifestyle groups, the National Health and Nutrition Examination Survey 2007 to 2018.

| **Demographic and health variable** | | **Prevalence of age-adjusted depression symptoms, weighted percentage (SE)** | **Estimated US population, n** | **Total** | **Lifestyle group^a^** | | | | ***P* value** |
| --- | --- | --- | --- | --- | --- | --- | --- | --- | --- |
|  |  |  |  |  | **Unhealthy diet and physically inactive** | **Healthy diet but physically inactive** | **Unhealthy diet but physically active** | **Healthy diet and physically active** |  |
|  | |  |  |  |  |  |  |  |  |
| Participants | | 7.08 (0.3) | 932,548,888 | 19,295 | 2565 | 915 | 11,560 | 4255 | N/A^b^ |
| **Age groups (years), mean (SD)** | | N/A | N/A | 45.74 (0.29) | 48.97 (0.42) | 55.18 (0.82) | 43.33 (0.33) | 48.56 (0.50) | <.001 |
|  | 20-39, n (weighted %) | N/A | 373,164,202 | 7332 (38.0) | 756 (30.7) | 163 (19.5) | 5090 (45.5) | 1323 (34.6) |  |
|  | 40-59, n (weighted %) | N/A | 349,230,849 | 6460 (33.4) | 890 (40.5) | 311 (41.6) | 3862 (36.7) | 1397 (37.1) |  |
|  | 60-80, n (weighted %) | N/A | 210,153,837 | 5503 (28.5) | 919 (28.9) | 441 (38.9) | 2608 (17.9) | 1535 (28.3) |  |
| **Sex, n (weighted %)** | | | | | | | | | <.001 |
|  | Male | 5.58 (0.3) | 480,853,104 | 10,147 (52.6) | 1065 (41.6) | 343 (32.6) | 6632 (56.4) | 2107 (47.7) |  |
|  | Female | 8.68 (0.4) | 451,695,784 | 9148 (47.4) | 1500 (58.4) | 572 (67.4) | 4928 (43.6) | 2148 (52.3) |  |
| **Race or ethnicity, n (weighted %)** | | | | | | | | | <.001 |
|  | Non-Hispanic White | 6.59 (0.4) | 645,679,595 | 8637 (44.8) | 1141 (68.8) | 346 (65.8) | 5253 (68.6) | 1897 (71.8) |  |
|  | Non-Hispanic Black | 8.78 (0.5) | 94,360,354 | 3930 (20.4) | 603 (11.7) | 170 (9.0) | 2468 (11.2) | 689 (6.8) |  |
|  | Mexican American | 7.96 (0.7) | 73,108,967 | 2654 (13.8) | 321 (7.0) | 129 (7.3) | 1664 (8.6) | 540 (6.3) |  |
|  | Other race (including multi-racial and other Hispanic) | 8.64 (0.7) | 119,399,972 | 4074 (21.1) | 500 (12.4) | 270 (17.9) | 2175 (11.6) | 1129 (15.1) |  |
| **Education, n (weighted %)** | | | | | | | | | <.001 |
|  | <9th grade | 11.44 (1.5) | 31,571,052 | 1381 (7.2) | 209 (4.1) | 90 (4.7) | 788 (3.4) | 294 (2.8) |  |
|  | 9-11th grade (including 12th grade with no diploma) | 12.71 (1.0) | 82,431,144 | 2373 (12.3) | 334 (10.1) | 105 (7.8) | 1606 (10.2) | 328 (5.0) |  |
|  | High school graduate or GED^c^ or equivalent | 8.30 (0.6) | 209,020,748 | 4348 (22.5) | 632 (25.0) | 168 (18.4) | 2861 (25.0) | 687 (15.3) |  |
|  | College graduate or above | 3.15 (0.4) | 304,559,557 | 5114 (26.5) | 587 (28.4) | 315 (43.5) | 2462 (26.7) | 1750 (48.2) |  |
|  | Some college or AA^d^ | 8.17 (0.6) | 304,966,388 | 6079 (31.5) | 803 (32.4) | 237 (25.7) | 3843 (34.8) | 1196 (28.7) |  |
| **Household income and PIR^e^, mean (SD)** | | N/A | N/A | 3.09 (0.04) | 3.02 (0.06) | 3.22 (0.09) | 2.93 (0.04) | 3.50 (0.05) | <.001 |
|  | Below poverty (<1.0), n (weighted %) | 15.90 (0.9) | 129,419,938 | 3859 (20.0) | 557 (14.8) | 150 (9.7) | 2557 (15.7) | 595 (9.4) |  |
|  | Above poverty (≥1.0), n (weighted %) | 5.73 (0.3) | 803,128,950 | 15,436 (80.0) | 2008 (85.17) | 765 (90.3) | 9003 (84.3) | 3660 (90.6) |  |
| **Marital status, n (weighted %)** | | | | | | | | | <.001 |
|  | Widowed or divorced or separated | 12.52 (0.9) | 155,234,085 | 3807 (19.7) | 640 (21.0) | 236 (20.5) | 2131 (16.4) | 800 (14.3) |  |
|  | Never married | 10.14 (1.0) | 193,116,412 | 3901 (20.2) | 415 (14.6) | 110 (13.3) | 2680 (23.7) | 696 (17.7) |  |
|  | Married or living with partner | 5.23 (0.3) | 584,198,392 | 11,587 (60.0) | 1510 (64.4) | 569 (66.3) | 6749 (59.9) | 2759 (68.0) |  |
| BMI (kg/m^2^), mean (SD) | | N/A | N/A | 28.73 (0.10) | 30.12 (0.21) | 28.74 (0.35) | 29.03 (0.10) | 27.23 (0.14) | <.001 |
| **Smoking status, n (weighted %)** | | | | | | | | | <.001 |
|  | Nonsmoker | 4.88 (0.3) | 518,650,675 | 10,680 (55.4) | 1375 (53.6) | 566 (57.3) | 6082 (53.6) | 2657 (61.5) |  |
|  | Former smoker | 5.96 (0.5) | 230,190,936 | 4633 (24.0) | 633 (24.8) | 253 (31.8) | 2590 (22.9) | 1157 (28.1) |  |
|  | Current smoker | 14.17 (0.9) | 183,707,278 | 3982 (20.6) | 557 (21.6) | 96 (10.9) | 2888 (23.5) | 441 (10.5) |  |
| **Alcohol use, n (weighted %)** | | | | | | | | | <.001 |
|  | Never | 5.72 (0.7) | 84,275,941 | 2251 (12.0) | 320 (9.6) | 187 (13.8) | 1197 (9.0) | 547 (9.1) |  |
|  | Former | 9.71 (0.9) | 97,695,182 | 2528 (13.5) | 450 (13.8) | 126 (13.4) | 1468 (10.4) | 484 (9.6) |  |
|  | Mild to moderate | 5.93 (0.3) | 511,607,553 | 9792 (52.3) | 1237 (55.2) | 474 (60.1) | 5627 (53.2) | 2454 (64.2) |  |
|  | Heavy | 8.16 (0.7) | 214,730,808 | 4154 (22.2) | 472 (21.4) | 102 (12.7) | 2925 (27.5) | 655 (17.1) |  |
| **Sleep time, n (weighted %)** | | | | | | | | | <.001 |
|  | <7 h | 5.26 (0.3) | 346,290,500 | 11,154 (57.9) | 1474 (62.9) | 538 (63.5) | 6423 (59.2) | 2719 (68.3) |  |
|  | 7 h≤time≤9 h | 10.06 (0.6) | 577,164,045 | 7935 (41.2) | 1063 (36.6) | 367 (36.0) | 5007 (40.0) | 1498 (31.0) |  |
|  | >9 h | 6.07 (1.6) | 9,094,344 | 172 (0.9) | 21 (0.60) | 9 (0.5) | 109 (1.2) | 33 (0.7) |  |
| **Whether taking antidepressant or anxiolytic medications, n (weighted %)** | | | | | | | | | <.001 |
|  | Yes | 4.55 (0.3) | 405,923,159 | 8672 (45.0) | 913 (34.4) | 271 (32.0) | 5706 (47.6) | 1782 (40.2) |  |
|  | No | 20.90 (1.3) | 110,528,053 | 1865 (9.7) | 337 (16.3) | 107 (13.9) | 1096 (11.5) | 325 (10.0) |  |
|  | Other | 6.41 (0.5) | 415,781,249 | 8750 (45.4) | 1314 (49.3) | 536 (54.1) | 4752 (40.9) | 2148 (49.9) |  |
| SB^f^ time, mean (SD) | | N/A | N/A | 357.36 (3.34) | 413.67 (6.17) | 402.13 (11.95) | 343.61 (3.29) | 355.08 (5.19) | <.001 |
| Moderate to vigorous PA^g^ (MET^h^ min/wk), mean (SD) | | N/A | N/A | 4890.89 (91.58) | 316.90 (4.24) | 330.58 (7.81) | 6306.99 (127.44) | 4476.12 (105.15) | <.001 |
| **Total energy intake, mean (SD)** | | | | 2126.75 (9.87) | 2029.80 (21.47) | 1881.95 (32.95) | 2191.62 (13.23) | 2061.22 (14.88) | <.001 |
|  | Tertile1 (<1661.167 kcal), n (weighted %) | 8.25 (0.6) | 254,652,757 | 5672 (33.3) | 857 (33.1) | 374 (40.9) | 3059 (28.5) | 1382 (33.2) |  |
|  | Tertile2 (1661.167-2312 kcal), n (weighted %) | 6.81 (0.5) | 285,728,980 | 5674 (33.4) | 777 (36.7) | 289 (36.8) | 3257 (33.1) | 1351 (36.2) |  |
|  | Tertile3 (>2312 kcal), n (weighted %) | 6.18 (0.4) | 288,475,532 | 5669 (33.3) | 650 (30.2) | 163 (22.3) | 3742 (38.4) | 1114 (30.6) |  |
| PHQ-9^i^ score, mean (SD) | | N/A | N/A | 2.89 (0.05) | 3.40 (0.12) | 3.02 (0.06) | 3.08 (0.19) | 2.28 (0.06) | <.001 |
| Total dietary quality score (HEI^j^-2015), mean (SD) | | N/A | N/A | 51.56 (0.27) | 44.87 (0.25) | 44.79 (0.18) | 68.75 (0.37) | 69.32 (0.19) | <.001 |
| **Survey cycle, n (weighted %)** | | | | | | | | | 0.11 |
|  | 2007-2008 | 6.57 (0.6) | 143,496,631 | 3278 (17.0) | 446 (16.4) | 146 (14.0) | 2009 (16.0) | 677 (13.6) |  |
|  | 2009-2010 | 7.07 (0.6) | 145,682,601 | 3458 (17.9) | 485 (16.7) | 160 (17.8) | 2059 (15.2) | 754 (15.9) |  |
|  | 2011-2012 | 6.41 (1.1) | 157,237,500 | 3099 (16.1) | 419 (17.4) | 162 (18.0) | 1779 (16.0) | 739 (18.5) |  |
|  | 2013-2014 | 7.56 (0.6) | 157,670,124 | 3338 (17.3) | 458 (18.2) | 180 (19.7) | 1934 (16.2) | 766 (17.6) |  |
|  | 2015-2016 | 7.23 (0.7) | 163,363,706 | 3133 (16.2) | 410 (16.0) | 142 (16.8) | 1895 (17.4) | 686 (18.7) |  |
|  | 2017-2018 | 7.39 (0.5) | 165,098,327 | 2989 (15.5) | 347 (15.3) | 125 (13.7) | 1884 (19.2) | 633 (15.8) |  |

^a^Lifestyle groups: unhealthy diet and physically inactive, participants did not meet the US PA recommendation guideline and below the 60th percentile of the HEI-2015 score; healthy diet but physically inactive, participants did not meet the US PA recommendation guideline but at or above the 60th percentile of the HEI-2015 score; unhealthy diet but physically active, participants met the US PA recommendation guideline but below the 60th percentile of the HEI-2015 score; healthy diet and physically active, participants met the US PA recommendation guideline and at or above the 60th percentile of the HEI-2015 score.

^b^N/A: not applicable.

^c^GED: general educational development.

^d^AA: Associate’s Degree.

^e^PIR: poverty income ratio.

^f^SB: sedentary behavior.

^g^PA: physical activity.

^h^MET: metabolic equivalent.

^i^PHQ-9: 9-Item Patient Health Questionnaire.

^j^HEI: Healthy Eating Index.
