# Supplementary material for: Association Between Joint Physical Activity and Dietary Quality and Lower Risk of Depression Symptoms in US Adults: Cross-sectional NHANES Study
Source: JMIR Public Health Surveill. 2023 May 10;9:e45776. doi: 10.2196/45776 (PMC10209797; doi:10.2196/45776)
Supplement: Multimedia Appendix 2 [file publichealth_v9i1e45776_app2.docx]

**Table S2** Characteristics of 19,295 US adults ≥ 20 years, stratified by depression symptoms status, the National Health and Nutrition Examination Survey 2007 to 2018.

| **Demographic and health variables** | | | | | | **Total** | **Depression symptoms status** | | ***P* value** |  |  |
| --- | --- | --- | --- | --- | --- | --- | --- | --- | --- | --- | --- |
|  |  |  |  |  |  |  | **No** | **Yes** |  |  |  |
|  | | | | | | | | | |  |  |
| **Age group (years), mean (SD)** | | | | | | 45.74 (0.29) | 45.85 (0.30) | 44.27 (0.66) | .02 |  |  |
|  | | | 20-39, n (weighted %) | | | 7332 (38.0) | 6752 (39.9) | 580 (41.5) |  |  |  |
|  | | | 40-59, n (weighted %) | | | 6460 (33.5) | 5883 (37.1) | 577 (42.2) |  |  |  |
|  | | | 60-80, n (weighted %) | | | 5503 (28.5) | 5153 (23.0) | 350 (16.3) |  |  |  |
| **Sex, n (weighted %)** | | | | | | | | |  |  |  |
|  | Male | | | | | 10,147 (52.6) | 9562 (52.4) | 585 (40.8) | <.001 | |  |
|  | Female | | | | | 9148 (47.4) | 8226 (47.6) | 922 (59.2) |  |  |  |
| **Race or ethnicity, n (weighted %)** | | | | | | | | | .002 |  |  |
|  | | Non-Hispanic White | | | | 8637 (44.7) | 7986 (69.7) | 651 (63.6) |  |  |  |
|  | | Non-Hispanic Black | | | | 3930 (20.4) | 3607 (9.9) | 323 (12.8) |  |  |  |
|  | | Mexican American | | | | 2654 (13.8) | 2447 (7.8) | 207 (8.1) |  |  |  |
|  | | Other race (including multi-racial, other Hispanic) | | | | 4074 (21.1) | 3748 (12.6) | 326 (15.5) |  |  |  |
| **Education, n (weighted %)** | | | | | | | | | <.001 |  |  |
|  | <9th Grade | | | | | 1381 (7.2) | 1229 (3.2) | 152 (5.3) |  | | |
|  | 9-11th Grade (Includes 12th grade with no diploma) | | | | | 2373 (12.3) | 2060 (8.3) | 313 (16) |  | | |
|  | High school graduate or GED^a^ or equivalent | | | | | 4348 (22.5) | 3980 (22.1) | 368 (26.3) |  | | |
|  | College graduate or above | | | | | 5114 (26.5) | 4932 (34.0) | 182 (14.5) |  | | |
|  | Some college or AA^b^ | | | | | 6079 (31.5) | 5587 (32.3) | 492 (38.0) |  | | |
| **Household income and PIR^c^**, **mean (SD)** | | | | | | 3.09 (0.04) | 3.16 (0.04) | 2.17 (0.07) | <.001 |  |  |
|  | Below poverty (< 1.0), n (weighted %) | | | | | 3859 (20.0) | 3296 (12.6) | 563 (30.5) |  |  |  |
|  | Above poverty (≥ 1.0), n (weighted %) | | | | | 15,436 (80.0) | 14,492 (87.4) | 944 (69.5) |  |  |  |
| **Marital status, n (weighted %)** | | | | | | | | | <.001 |  |  |
|  | | | | Widowed or divorced or separated | | 3807 (19.7) | 3342 (15.9) | 465 (26.9) |  |  |  |
|  | | | | Never married | | 3901 (20.2) | 3528 (20.2) | 373 (27.2) |  |  |  |
|  | | | | Married or living with partner | | 11,587 (60.1) | 10,918 (63.9) | 669 (46.0) |  |  |  |
| BMI (kg/m2), mean (SD) | | | | | | 28.73 (0.10) | 28.62 (0.10) | 30.15 (0.28) | <.001 |  |  |
| **Smoking status, n (weighted %)** | | | | | | | | | <.001 |  |  |
|  | Nonsmoker | | | | | 10,680 (55.4) | 10,085 (56.9) | 595 (38.6) |  | |  |
|  | Former smoker | | | | | 4633 (24.0) | 4307 (25.0) | 326 (20.8) |  | |  |
|  | Current smoker | | | | | 3982 (20.6) | 3396 (18.1) | 586 (40.6) |  | |  |
| **Alcohol use, n (weighted %)** | | | | | | | | | <.001 |  |  |
|  | | Never | | | | 2251 (12.0) | 2100 (9.4) | 151 (7.6) |  |  |  |
|  | | Former | | | | 2528 (13.5) | 2266 (10.4) | 262 (15.7) |  |  |  |
|  | | Mild-to-moderate | | | | 9792 (52.3) | 9170 (57.0) | 622 (47.5) |  |  |  |
|  | | Heavy | | | | 4154 (22.2) | 3743 (23.2) | 411 (29.2) |  |  |  |
| **Lifestyle group^d^, n (weighted %)** | | | | | | | | | <.001 |  |  |
|  | | | | | Unhealthy diet and physically inactive | 2565 (13.3) | 2301 (12.1) | 264 (16.4) |  |  |  |
|  | | | | | Healthy diet but physically inactive | 915 (4.7) | 833 (3.9) | 82 (4.8) |  |  |  |
|  | | | | | Unhealthy diet but physically active | 11,560 (59.9) | 10,599 (59.5) | 961 (64.9) |  |  |  |
|  | | | | | Healthy diet and physically active | 4255 (22.1) | 4055 (24.4) | 200 (13.9) |  |  |  |
| **Sleep time, n (weighted %)** | | | | | | | | | <.001 |  |  |
|  | | | <7 h | | | 11,154 (57.8) | 10,526 (63.1) | 628 (45.8) |  |  |  |
|  | | | 7 h≤time≤9 h | | | 7969 (41.3) | 7102 (35.90) | 867 (53.3) |  |  |  |
|  | | | >9 h | | | 172 (0.9) | 160 (1.0) | 12 (1.0) |  |  |  |
| **Whether taking antidepressant or anxiolytic medications, n (weighted %)** | | | | | | | | | <.001 |  |  |
|  | | | | Yes | | 8672 (45.0) | 8192 (44.6) | 480 (29.6) |  |  |  |
|  | | | | No | | 1865 (9.7) | 1416 (10.2) | 449 (33.8) |  |  |  |
|  | | | | Other | | 8750 (45.4) | 8172 (45.2) | 578 (36.6) |  |  |  |
| SB^e^ time, mean (SD) | | | | | | 357.36 (3.34) | 356.68 (3.43) | 366.30 (8.97) | .29 |  |  |
| Moderate to vigorous PA^f^ (MET^g^ min/wk), mean (SD) | | | | | | 4890.89 (91.58) | 4917.60 (96.96) | 4541.00 (238.20) | .15 |  |  |
| **Total energy intake, mean (SD)** | | | | | | 2126.75 (9.87) | 2133.87 (10.43) | 2032.24 (28.57) | .001 |  |  |
|  | | Tertile1(< 1661.167 kcal), n (weighted %) | | | | 5672 (33.3) | 5183 (30.4) | 489 (35.5) |  |  |  |
|  | | Tertile2(1661.167-2312 kcal), n (weighted %) | | | | 5674 (33.4) | 5,245 (34.6) | 429 (33.2) |  |  |  |
|  | | Tertile3(> 2312 kcal), n (weighted %) | | | | 5669 (33.3) | 5,259 (35.1) | 410 (31.3) |  |  |  |
| PHQ-9^h^ Score, mean (SD) | | | | | | 2.89 (0.05) | 2.05 (0.03) | 14.00 (0.14) | <.001 |  |  |
| Total dietary quality score (HEI^i^-2015), mean (SD) | | | | | | 51.56 (0.27) | 51.83 (0.27) | 47.95 (0.53) | <.001 |  |  |
| **Survey cycle, n (weighted %)** | | | | | | | | | .86 |  |  |
|  | | | | 2007-2008 | | 3278 (17.0) | 3021 (15.4) | 257 (14.5) |  |  |  |
|  | | | | 2009-2010 | | 3458 (17.9) | 3168 (15.6) | 290 (15.0) |  |  |  |
|  | | | | 2011-2012 | | 3099 (16.1) | 2869 (17.0) | 230 (15.3) |  |  |  |
|  | | | | 2013-2014 | | 3338 (17.3) | 3088 (16.8) | 250 (18.0) |  |  |  |
|  | | | | 2015-2016 | | 3133 (16.2) | 2897 (17.5) | 236 (17.8) |  |  |  |
|  | | | | 2017-2018 | | 2989 (15.5) | 2745 (17.7) | 244 (18.4) |  |  |  |

^a^GED: general educational development.

^b^AA: Associate's Degree.

^c^PIR, Poverty income ratio.

^d^Lifestyle groups: Unhealthy diet and physically inactive, participants did not meet the US PA recommendation guideline and below the 60th percentile of the HEI-2015 score; Healthy diet but physically inactive, participants did not meet the US PA recommendation guideline but at or above the 60th percentile of the HEI-2015 score; Unhealthy diet but physically active, participants met the US PA recommendation guideline but below the 60th percentile of the HEI-2015 score; Healthy diet and physically active, participants met the US PA recommendation guideline and at or above the 60th percentile of the HEI-2015 score.

^e^SB: sedentary behaviour.

^f^PA: physical activity.

^g^MET: metabolic equivalent.

^h^PHQ-9: 9-Item Patient Health Questionnaire.
^i^HEI: Healthy Eating Index.
