# Supplementary material for: Association Between Joint Physical Activity and Dietary Quality and Lower Risk of Depression Symptoms in US Adults: Cross-sectional NHANES Study
Source: JMIR Public Health Surveill. 2023 May 10;9:e45776. doi: 10.2196/45776 (PMC10209797; doi:10.2196/45776)
Supplement: Multimedia Appendix 3 [file publichealth_v9i1e45776_app3.docx]

**Table S3** Characteristics of 19,295 US adults ≥ 20 years, stratified by survey cycle, the National Health and Nutrition Examination Survey 2007 to 2018.

| **Demographic and health variable** | | | | **Total** | **Survey cycle** | | | | | | ***P* value** |
| --- | --- | --- | --- | --- | --- | --- | --- | --- | --- | --- | --- |
|  |  |  |  |  | **2007-2008** | **2009-2010** | **2011-2012** | **2013-2014** | **2015-2016** | **2017-2018** |  |
|  | | | | | | | | | | | |
| No. of participants | | | | 19,295 | 3278 | 3458 | 3099 | 3338 | 3133 | 2989 |  |
| Prevalence of age-adjusted depression symptoms | | | |  | 6.57 (0.6) | 7.07 (0.6) | 6.41 (1.1) | 7.56 (0.6) | 7.23 (0.7) | 7.39 (0.5) |  |
| **Age group (years), mean (SD)** | | | | 45.74 (0.29) | 44.63 (0.65) | 45.37 (0.54) | 45.41 (1.00) | 45.90 (0.51) | 46.31 (0.70) | 46.64 (0.77) | .36 |
|  | | 20-39, n (weighted %) | | 7332 (38.0) | 1239 (41.0) | 1302 (39.1) | 1277 (40.3) | 1283 (39.9) | 1205 (39.6) | 1026 (39.5) | .14 |
|  | | 40-59, n (weighted %) | | 6460 (33.5) | 1065 (38.8) | 1222 (40.0) | 997 (37.8) | 1143 (36.2) | 1052 (36.3) | 981 (35.2) |  |
|  | | 60-80, n (weighted %) | | 5503 (28.5) | 974 (19.3) | 934 (19.9) | 825 (21.0) | 912 (23.9) | 876 (24.2) | 982 (25.3) |  |
| **Sex, n (weighted %)** | | | |  |  |  |  |  |  |  | .74 |
|  | | Male | | 10,147 (52.6) | 1760 (50.2) | 1806 (51.8) | 1661 (52.3) | 1729 (52.1) | 1641 (51.2) | 1550 (51.6) |  |
|  | | Female | | 9148 (47.4) | 1518 (49.8) | 1652 (48.2) | 1438 (47.7) | 1609 (47.9) | 1492 (48.8) | 1439 (48.4) |  |
| **Race or ethnicity, n (weighted %)** | | | |  |  |  |  |  |  |  | .51 |
|  | | Non-Hispanic White | | 8637 (44.8) | 1690 (73.7) | 1831 (73.2) | 1277 (70.0) | 1517 (67.0) | 1176 (68.0) | 1146 (64.5) |  |
|  | | Non-Hispanic Black | | 3930 (20.4) | 599 (9.4) | 575 (9.8) | 780 (10.3) | 660 (11.0) | 660 (9.8) | 656 (10.2) |  |
|  | | Mexican American | | 2654 (13.8) | 538 (7.8) | 561 (7.1) | 277 (6.8) | 412 (8.6) | 490 (8.0) | 376 (8.6) |  |
|  | | Other race (including multi-racial, other Hispanic) | | 4074 (21.1) | 451 (9.0) | 491 (9.9) | 765 (12.9) | 749 (13.5) | 807 (14.1) | 811 (16.7) |  |
| **Education, n (weighted %)** | | | |  |  |  |  |  |  |  | .003 |
|  | | | <9th Grade | 1381 (7.2) | 318 (4.8) | 302 (4.0) | 185 (2.9) | 159 (2.8) | 248 (3.6) | 169 (2.4) |  |
|  | | | 9-11th Grade (Includes 12th grade with no diploma) | 2373 (12.3) | 527 (12.0) | 478 (10.6) | 367 (8.7) | 405 (9.7) | 304 (6.4) | 292 (6.3) |  |
|  | | | High school graduate or GED^a^ or equivalent | 4348 (22.5) | 785 (24.2) | 787 (21.6) | 618 (19.0) | 738 (21.0) | 696 (20.5) | 724 (28.1) |  |
|  | | | College graduate or above | 5114 (26.5) | 720 (27.9) | 841 (31.7) | 925 (35.4) | 951 (33.3) | 884 (35.1) | 793 (32.0) |  |
|  | | | Some college or AA^b^ | 6079 (31.5) | 928 (31.1) | 1050 (32.2) | 1004 (33.9) | 1085 (33.1) | 1001 (34.4) | 1011 (31.3) |  |
| **Household income and PIR^c^,** **mean (SD)** | | | | 3.09 (0.04) | 3.16 (0.10) | 3.14 (0.04) | 2.99 (0.12) | 3.01 (0.12) | 3.12 (0.10) | 3.14 (0.07) | .78 |
| Below poverty (< 1.0), n (weighted %) | | | | 3859 (20.0) | 617 (13.3) | 718 (13.9) | 698 (16.2) | 678 (14.3) | 644 (13.5) | 504 (12.1) | .56 |
| Above poverty (≥ 1.0), n (weighted %) | | | | 15,436 (80.0) | 2661 (86.7) | 2740 (86.1) | 2401 (83.8) | 2660 (85.7) | 2489 (86.5) | 2485 (87.9) |  |
| **Marital status, n (weighted %)** | | | |  |  |  |  |  |  |  | .59 |
|  | Widowed or divorced or separated | | | 3807 (19.7) | 655 (16.0) | 685 (16.5) | 603 (17.5) | 661 (17.8) | 595 (15.7) | 608 (16.4) |  |
|  | Never married | | | 3901 (20.2) | 594 (20.4) | 668 (20.1) | 769 (23.4) | 679 (21.1) | 617 (18.4) | 574 (20.9) |  |
|  | Married or living with partner | | | 11,587 (60.1) | 2029 (63.6) | 2105 (63.3) | 1727 (59.1) | 1998 (61.2) | 1921 (65.9) | 1807 (62.8) |  |
| BMI (kg/m2), mean (SD) | | | | 28.73 (0.10) | 28.24 (0.19) | 28.40 (0.17) | 28.23 (0.20) | 28.55 (0.19) | 29.18 (0.31) | 29.62 (0.26) | <.001 |
| **Smoking status, n (weighted %)** | | | |  |  |  |  |  |  |  | .21 |
|  | Nonsmoker | | | 10,680 (55.4) | 1700 (51.8) | 1857 (55.4) | 1776 (55.5) | 1893 (56.9) | 1763 (55.8) | 1691 (57.8) |  |
|  | Former smoker | | | 4633 (24.0) | 829 (24.6) | 840 (24.4) | 702 (24.6) | 762 (23.9) | 755 (26.0) | 745 (24.6) |  |
|  | Current smoker | | | 3982 (20.6) | 749 (23.6) | 761 (20.2) | 621 (19.9) | 683 (19.2) | 615 (18.2) | 553 (17.6) |  |
| **Alcohol use, n (weighted %)** | | | |  |  |  |  |  |  |  | <.001 |
|  | | Never | | 2251 (12.0) | 405 (9.8) | 350 (8.3) | 383 (8.6) | 430 (11.1) | 419 (10.1) | 264 (7.6) |  |
|  | | Former | | 2528 (13.5) | 584 (14.4) | 531 (12.7) | 466 (12.3) | 506 (12.5) | 441 (11.9) | 0 (0) |  |
|  | | Mild-to-moderate | | 9792 (52.3) | 1564 (52.3) | 1701 (54.1) | 1577 (54.2) | 1717 (55.3) | 1604 (55.7) | 1629 (66.9) |  |
|  | | Heavy | | 4154 (22.2) | 718 (23.6) | 870 (24.8) | 666 (24.8) | 681 (21.2) | 664 (22.3) | 555 (25.6) |  |
| **Sleep time, n (weighted %)** | | | |  |  |  |  |  |  |  | <.001 |
|  | | <7 h | | 11,154 (57.8) | 1784 (57.7) | 1856 (57.5) | 1695 (57.5) | 1813 (58.5) | 2100 (72.3) | 1906 (66.6) |  |
|  | | 7 h≤time≤9 h | | 7969 (41.3) | 1494 (42.3) | 1602 (42.6) | 1404 (42.5) | 1525 (41.5) | 945 (25.0) | 999 (30.6) |  |
|  | | >9 h | | 172 (0.9) | 0 | 0 | 0 | 0 | 88 (2.8) | 84 (2.8) |  |
| **Whether taking antidepressant or anxiolytic medications, n (weighted %)** | | | |  |  |  |  |  |  |  | .81 |
|  | | Yes | | 1865 (9.7) | 331 (12.2) | 295 (10.2) | 276 (11.6) | 334 (12.2) | 291 (11.5) | 338 (13.3) |  |
|  | | No | | 8672 (45.0) | 1454 (43.1) | 1593 (44.6) | 1423 (43.1) | 1529 (43.4) | 1420 (44.3) | 1253 (42.8) |  |
|  | | other | | 8750 (45.4) | 1492 (44.73) | 1569 (45.2) | 1399 (45.3) | 1474 (44.4) | 1420 (44.2) | 1396 (43.9) |  |
| SB^d^ time, mean (SD) | | | | 357.36 (3.34) | 315.21 (8.16) | 336.22 (6.91) | 364.48 (7.85) | 409.56 (7.96) | 378.16 (7.84) | 335.45 (8.12) | <.001 |
| Moderate to vigorous PA^e^ (MET^f^ minutes/week), mean (SD) | | | | 4890.89 (91.58) | 5222.22 (229.12) | 4354.52 (178.50) | 4298.60 (177.50) | 4409.80 (186.08) | 5088.97 (222.70) | 5903.73 (303.81) | <.001 |
| **Total energy intake**, **mean (SD)** | | | | 2126.75 (9.87) | 2136.65 (24.98) | 2160.25 (21.04) | 2132.92 (20.21) | 2139.50 (27.00) | 2075.14 (23.78) | 2119.51 (26.80) | .18 |
|  | | | Tertile1(< 1661.167 kcal), n (weighted %) | 5672 (33.3) | 1014 (32.1) | 1020 (29.5) | 871 (28.7) | 977 (30.2) | 840 (30.3) | 950 (33.4) | .07 |
|  | | | Tertile2(1661.167-2312 kcal), n (weighted %) | 5674 (33.4) | 928 (33.4) | 1011 (35.2) | 951 (35.3) | 1018 (35.4) | 965 (36.6) | 801 (31.0) |  |
|  | | | Tertile3(> 2312 kcal), n (weighted %) | 5669 (33.3) | 919 (34.4) | 1034 (35.4) | 962 (36.0) | 1000 (34.4) | 845 (33.1) | 909 (35.5) |  |
| PHQ-9^g^ Score, mean (SD), n (weighted %) | | | | 2.89 (0.05) | 2.89 (0.10) | 2.93 (0.14) | 2.70 (0.18) | 2.90 (0.10) | 2.98 (0.10) | 2.95 (0.08) | .85 |
| Total dietary quality score (HEI^h^-2015), mean (SD) | | | | 51.56 (0.27) | 50.66 (0.87) | 52.23 (0.30) | 52.74 (0.57) | 52.03 (0.57) | 51.72 (0.68) | 50.01 (0.78) | .05 |

^a^GED: general educational development.

^b^AA: Associate's Degree.

^c^PIR, Poverty income ratio.

^d^SB: sedentary behaviour.

^e^PA: physical activity.

^f^MET: metabolic equivalent.

^g^PHQ-9, 9-Item Patient Health Questionnaire.

^h^HEI: Healthy Eating Index.
