# Supplementary material for: Association Between Joint Physical Activity and Dietary Quality and Lower Risk of Depression Symptoms in US Adults: Cross-sectional NHANES Study
Source: JMIR Public Health Surveill. 2023 May 10;9:e45776. doi: 10.2196/45776 (PMC10209797; doi:10.2196/45776)
Supplement: Multimedia Appendix 4 [file publichealth_v9i1e45776_app4.docx]

**Table S4** Association between lifestyle and risk of depression symptoms with the exclusion of extreme poverty-to-income ratio (PIR), BMI values, and adjustment for alcohol use, total energy intake, and use of antidepressant or anxiolytic medications.

| **Variables** | **Met PA**^a^ | | **Higher dietary quality**^b^ | | **Healthy diet but physically inactive**^c^ | | **Unhealthy diet but physically active**^d^ | | **Healthy diet and physically active**^e^ | |
| --- | --- | --- | --- | --- | --- | --- | --- | --- | --- | --- |
|  | Adjusted OR (95%CI) | *P* Value | Adjusted OR (95%CI) | *P* Value | Adjusted OR (95%CI) | *P* Value | Adjusted OR (95%CI) | *P* Value | Adjusted OR (95%CI) | *P* Value |
|  |  |  |  |  |  |  |  |  |  |  |
| Excluding extreme PIR^f^ values | 0.798 (0.697, 0.916) | .001 | 0.819 (0.708, 0.945) | .007 | 1.066 (0.807, 1.396) | .65 | 0.863 (0.740, 1.008) | .06 | 0.647 (0.528, 0.793) | <.001 |
| Excluding extreme BMI^g^ values | 0.818 (0.715, 0.938) | .004 | 0.824 (0.713, 0.948) | .008 | 1.066 (0.807, 1.395) | .65 | 0.882 (0.758, 1.030) | .11 | 0.669 (0.547, 0.817) | <.001 |
| Alcohol user (former, never and now) | 0.793 (0.692,0.912) | .001 | 0.864 (0.746,0.996) | .046 | 1.093 (0.825,1.435) | .53 | 0.850 (0.728,0.995) | .04 | 0.681 (0.555,0.834) | <.001 |
| Total energy intake | 0.833 (0.722,0.963) | .01 | 0.800 (0.686,0.930) | .004 | 0.917 (0.675,1.230) | .57 | 0.866 (0.737,1.019) | .08 | 0.665 (0.538,0.821) | <.001 |
| Whether taking antidepressant or anxiolytic medications | 0.844 (0.736, 0.971) | .02 | 0.841 (0.727, 0.971) | .019 | 1.078 (0.812,1.419) | .60 | 0.908 (0.777, 1.064) | .23 | 0.704 (0.573, 0.863) | <.001 |

^a^Met PA: participants met the US PA guideline.

^b^Higher dietary quality: participants who scored at or above the 60th percentile on the HEI.

^c^Healthy diet but physically inactive: participants did not meet the US PA recommendation guideline but at or above the 60th percentile of the HEI-2015 score.

^d^Unhealthy diet but physically active: participants met the US PA recommendation guideline but below the 60th percentile of the HEI-2015 score.

^e^Healthy diet and physically active: participants met the US PA recommendation guideline and at or above the 60th percentile of the HEI-2015 score.
^f^PIR: Poverty income ratio;

^g^BMI: body mass index.

Multivariable model was adjusted for age, sex, race or ethnicity, education, marital status, BMI, PIR, smoking status, SB and sleep time.
